# Central Park Menagerie—Autopsy of Kangaroo

**Published:** 1890-01

**Authors:** 


### CASES, SELECTIONS, TRANSLATIONS.

### CENTRAL PARK MENAGERIE—AUTOPSY—KANGAROO.

*Petrogale-penicillata;* brush-tailed rock kangaroo. The animal apparently in the best of health, fell off a bench while playing, and was instantly killed. Bloody suffusion of the conjunctiva of the right eye was noted. The dissection was made five hours after death, November 21st. Only the brain and spinal cord being examined. Inasmuch as the various species of kangaroo are characterized by an enormous development of the hind limbs and tail, a comparison of the various dimensions of the cord becomes most interesting. The following measurements of the entire animal will aid in forming a conception of the relative development of the brain and cord :—

Weight, fourteen pounds ; total length from tip of snout to tip of tail measured while the animal had stiffened in its natural attitude, was exactly 39 inches. Of this 20 appertain to the tail and 19 to the remainder of the body. The anterior extremity (shoulder joint to end of claw) was 6, the posterior (hip joint to end of claw) 13 inches long. The girth of the thorax, behind the fore-limb attachment was 12, that of the abdomen 19 inches.

The spinal canal undergoes a remarkable incurvation with its concavity dorsad between the ilia. The consequence is that sinking so deeply between their large bony elevation and beneath the dorsal muscles, it is very diffiult to remove it in the ordinary way. The cord closely corresponds in length to the containing canal. The conus tapers evenly from a base which bears the proportion to the length of 1:7. There is no filum in the sense of an abruptly demarked thin end of the conus. The cervical enlargement is 8.5 mm. wide, and 6. mm. deep. The lumbar is 11.5 mm. wide and 9 mm. deep.

The lumbar enlargement tapers more rapidly (losing three millimeters in transverse diameter within a length of twelve millimeters) to a point which is five centimeters from the end of the conus, towards which there is an even tapering as elsewhere mentioned.

The length of the lumbar enlargement (upper boundary uncertain) is 6 centimeters, of the cervical 3-4 centimeters.

As far as can be seen with the naked eye the posterior columnas and posterior cornua of gray matter are comparatively small, reminding one, very much of the similar relation found in the rabbit.

The total length of the cord is 14¾ inches (32.05 centimeters), reaching to within 3¼ inches (8.2 centimeters) of the root of the tail. The total weight of the central nervous system is 33.5 grammes, of which 18.5 grammes appertains to the brain, and 15 to the cord.

The skull was broken in two places, both exhibiting vitreous fracture· The anterior was represented by a linear crack bent at an obtuse angle, and marked by a dark blood line. This blood came from the torn superior longitudinal sinus. The other in the occipital crest was a stellated fracture. Underneath the former, there was an enormous sub-dural hemorrhage which had not alone flattened the underlyiug right cerebral hemisphere, but also crowded the left lateral of the median line. The interior of the brain has not yet been examined. The blood had forced its way into the spinal dural sac, and (through as yet unstudied channels[1] )into the canalis centralis of the cord. Everywhere a cross section of the cord even down to the *conus terminalis* revealed a central clot, distending the canal to its utmost. Future study of microscopic sections may reveal the existence of a communication with some space coelal or lymphatic in the post septum thus demonstrated by this natural injection. E. C. S

